# Kuntscher Nail: A Forgotten Entity Yet a Reliable Modality in Treatment of Winquist Type I and II Closed Femoral Shaft Fractures

**DOI:** 10.7759/cureus.10608

**Published:** 2020-09-23

**Authors:** Ajay Bharti, Sanjay Kumar, Sudhir Shyam Kushwaha, Anil Kumar Gupta, Nitish Kumar, Atil K Lal

**Affiliations:** 1 Department of Orthopedics, All India Institute of Medical Sciences, Gorakhpur, IND; 2 Department of Orthopedics, Ganesh Shankar Vidyarthi Memorial Medical College, Kanpur, IND; 3 Department of Orthopedics, Government Medical College Azamgarh, Azamgarh, IND

**Keywords:** kuntscher nailing, interlocking nailing, winquist type i and ii, femoral shaft fractures

## Abstract

Background

Interlocking intramedullary nail is used for almost all types of femoral shaft fractures worldwide because of its better mechanical stability. In countries like India with limited healthcare infrastructure, the use of Kuntscher nail (K-nail) in Winquist type I and type II isthmic fractures is still debated. Therefore, we conducted this study to compare the functional outcome, radiation exposure, and cost of the implant between closed reduction and internal fixation of Winquist type I and II fractures in the middle one-third shaft region by K-nail and intramedullary locked nails (IMILN), respectively.

Methods

This was a hospital-based non-blinded randomized trial which included 56 patients with closed Winquist type I and II femoral shaft fractures of the middle one-third^ ^femoral shaft (isthmic and paraisthmic zone). All the patients were either treated by K-nail or IMILN. The patients were followed up every three weeks for the initial six months and quarterly thereafter. Per operative duration of surgery and radiation exposure by C-arm was documented and assuming scattered radiation 20%, radiation exposure to the surgeon was calculated and patients were assessed clinically and radiologically for union.

Results

The patients were divided into two groups: group I (K-nail, n = 25) and group II (IMILN, n = 31). In groups I and II, the patients achieving radiological union were 88% (n = 22/25) and 84% (n = 27/31) at six months’ duration, respectively. The mean duration for the radiological union was 3.65 ± 0.55 months in group I (K-nail) and 3.76 ± 0.59 months in group II (IMILN), respectively. There was no statistically significant difference in the duration of the union (p = 0.4963). The average number of C- arm exposures was 16.36 ± 3.18 in group I as compared to 27.29 ± 4.01 in group II, and the mean scattered radiation was 5.0 ± 1.63 in group I and 6.61 ± 4.06 in group II. This difference was statistically significant.

Conclusion

Kuntscher intramedullary nailing can provide comparable rates of union as is achieved with interlocking intramedullary nailing with an advantage of less radiation exposure and duration of surgery, provided the patient selection is proper (isthmic and paraisthmic zone).

## Introduction

Femoral shaft fractures are one of the commonest high energy fractures, and intramedullary nailing has now become the gold standard management of these fractures [1,2]. Intramedullary fixation is considered superior to plate fixation because of lower rates of infection, early weight-bearing, and lesser chances of non-union [3]. Gerhard Kuntscher in 1940 first introduced the cloverleaf nail for the intramedullary internal fixation of femoral shaft fractures [4]. Later, various studies [5,6] proved that unlocked Kuntscher nail (K-nail) should only be used for isthmic femoral shaft fractures of Winquist type I and type II. The main drawback of K-nail for use in other types of femoral shaft fracture was lack of rotational stability. Nowadays, interlocking intramedullary nail is used for almost all types of femoral shaft fractures in developed countries because of better rotational stability.

However, in countries like India with limited healthcare infrastructure, the use of K-nail in Winquist type I and type II isthmic fractures is still debated. The reason for this includes lesser duration of the surgery, cost of the implant, lesser radiation exposure, or the procedure can be done without image intensifier also and has a comparable functional outcome with an interlocking nail (ILN) in these types of fractures.

We, therefore, conducted this study to compare the functional outcome, radiation exposure, and cost of the implant between closed reduction and internal fixation of Winquist [7] type I and II fractures in the middle one-third shaft region by K-nail and intramedullary locked nails (IMILN), respectively.

## Materials and methods

Study design

This is a hospital-based non-blinded randomized trial conducted in the Medical College of Northern India.

Participants

Patients between the age of 18 and 55 years with closed Winquist type I and II femoral shaft fractures of the middle one-third femoral shaft (isthmic and paraisthmic zone) reporting to the emergency department were included in the study. Patients with medical comorbidities, pathological fracture, fractures with vascular injury, open fractures, and those cases in which open reduction was needed were excluded from the study.

Study duration and sample size

Due to the variable number of patients reporting to the emergency with Winquist type I and II femoral shaft fractures of the middle one-third femoral shaft (isthmic and paraisthmic zone), no sample size was calculated. Instead, a study duration of three years was fixed, including 18 months of enrolment period (December 2013 and May 2015).

Methodology

All patients reporting to the emergency department were assessed for the nature and type of fracture according to a fixed protocol of the Institute. After diagnosis and written informed consent, above-mentioned patients were included in the study. Those participants who agreed to participate in the study were randomly allocated to either one of the intervention groups stated above by concealed envelopes using the block randomization method.

Intervention 

All the selected patients underwent closed reduction and internal fixation with intramedullary K-nailing (IMKN) group I and intramedullary interlocking nailing (IMILN) group II, as per the standard technique [7].

Follow-up of patients

Routinely all the patients were followed up every three weeks for an initial six months and quarterly thereafter. On each visit, patients were assessed clinically and radiologically for union and complications, if any. Partial weight-bearing was started in group I cases only after radiological evidence of bridging callus formation and full weight-bearing after fracture union. In group II, partial weight-bearing was started as soon as pain permitted followed by full weight-bearing after fracture union. The functional outcome was also assessed using the modified Harris hip score [8] and Knee society score [9].

Outcome

The mean duration of union (in months) was considered as the primary outcome. The functional outcome measured at nine months was considered as a secondary outcome.

Study definitions

The radiological union was defined as the presence of bridging callus across the fracture site in at least three cortices in both anteroposterior (AP) and lateral X-ray images [10].

The fracture was considered as healed or consolidated when there was complete radiological union and the patient was able to bear weight on his operated limb without pain at the fracture site [10].

The fractures were categorized in state of delayed union which took more than six months for union and those fractures in which there was an absence of callus at six months were in a state of non-union [6].

Statistical analysis

Data were entered in Microsoft excel and analysis was done using SPSS version 22 (Armonk, NY: IBM Corp). T statistics for continuous data and chi-square test for categorical data were used, and p values of less than 0.05 were considered statistically significant. Per protocol, analysis was performed. The functional outcome was also assessed using the modified Harris hip score and Knee society score according to the standard methods.

Approval was taken from the Institutional review board prior to the study (IEC/RC/Ortho/ 02/Thesis/Oct2013). Written informed consent was obtained from all the participants prior to the study. Confidentiality of the patient data was maintained throughout the study period.

## Results

At the end of one and half year, 68 cases were enrolled in the study; out of which 9 cases were lost to follow-up and 3 cases required open reduction (group I); therefore, finally we had total 56 cases as per our inclusion criteria with an average follow-up of 18.08 months (range, 9-36 months).

Either of the two surgical procedures was performed on the patients as per simple random allocation. There were 25 patients in group I, who were managed by IMKN, and 31 patients in group II, who were managed by IMILN. The mean age of the patients was 34.28 years. There was no significant difference between both the groups in terms of sex and percentage of fracture type.

**Figure 1:**
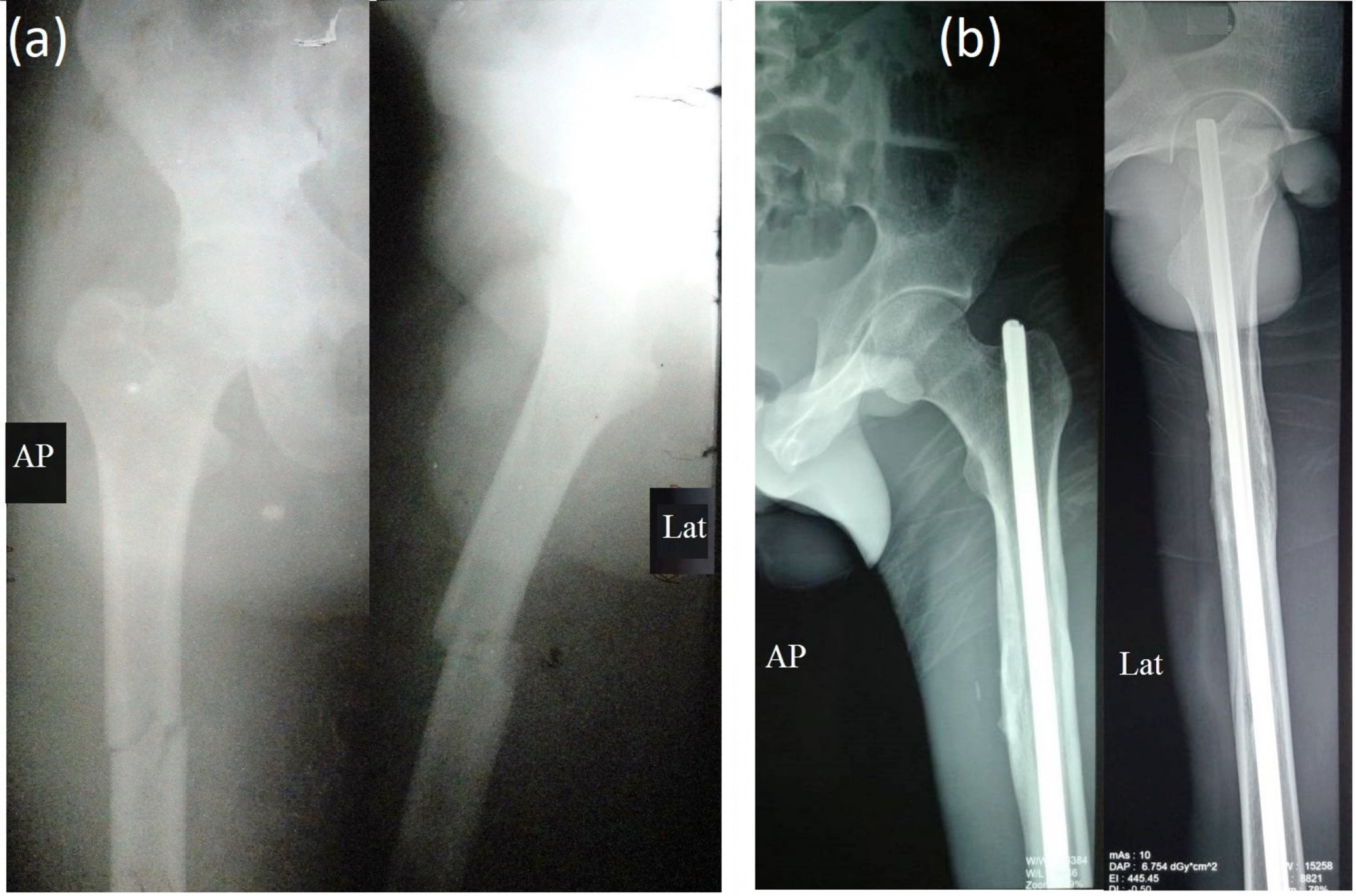
(a) Preop X-ray. (b) Postop X-ray at three years. Follow-up of a case managed with K-nailing (group I)

**Figure 2:**
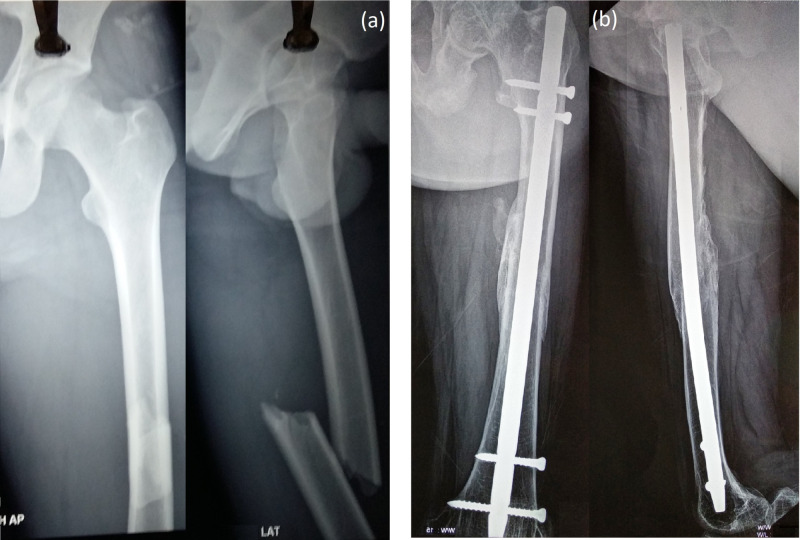
(a) Preop X-ray. (b) Postop X-ray at 22 months. Follow-up of a case managed with interlocking nailing (group II)

In groups I and II, the patients achieving radiological union were 88% (n = 22/25) and 84% (n = 27/31) at six months’ duration, respectively. The mean duration for radiological union was 3.65 ± 0.55 months in group I (K-nail) and 3.76 ± 0.59 months in group II (IMILN), respectively (Table 1; Figures 1, 2).

**Table 1:** Status of union and full weight-bearing in both groups of nailing

Procedure	No. of cases	Mean duration for union (months)	Mean duration for full weight-bearing (months)
Group I ( K-nailing)	25	3.65 ± 0.55	4.64 ± 0.54
Group II (interlocking nailing)	31	3.76 ± 0.59	4.63 ± 0.60
P value		0.478	0.949

There was no statistically significant difference in the duration of union (p = 0.4963). Functional outcome was assessed by the modified Harris hip score and Knee Society Score (p = 0.8316 and 0.525) between the groups; both groups had more than 90% of its scores in the excellent to good range (Table 2).

**Table 2:** Functional outcome at latest follow-up with an average follow-up of 18.08 months (range 9-36 months) by the modified Harris hip score and Knee Society score

Modified Harris hip score	Group I ( K-nailing)	Group II (interlocking nailing)
Excellent, >90	88% (22)	91% (28)
Good, 80-89	08% (2)	03% (1)
Satisfactory, 70-79	04% (1 )	06% (2)
Poor, <70	00	00
Knee Society score		
Excellent, 80-100	93% (23)	87% (27)
Good, 70-79	00	03% (1)
Fair, 60-69	08% (2)	10% (3)
Poor, <60	00	00

The mean duration of surgery was lower in group I (36.80 ± 3.18) as compared to group II (50.32 ± 7.12), the average number of C-arm exposures was 16.36 ± 3.18 in group I as compared to 27.29 ± 4.01 in group II, and mean scattered radiation was 5.0 ± 1.63 in group I and 6.61 ± 4.06 in group II. All these differences were statistically significant (Table 3).

**Table 3:** Per operative C-arm exposure and duration of surgery

Intervention groups	No. of cases	Mean duration of surgery in minutes	Average number of C-arm radiation exposure	Mean duration of C-arm radiation exposure in seconds	Mean C-arm radiation emitted in millisievert (mSv)	Mean C-arm scattered radiation exposure to surgeon in millisievert (mSv)
Group I (K-nailing)	25	36.80 ± 3.18	16.36 ± 3.18	54 ± 17.38	25.18 ± 8.12	5.0 ± 1.63
Group II (interlocking nailing)	31	50.32 ± 7.12	27.29 ± 4.01	71.29 ± 43.49	33.25 ± 20.30	6.61 ± 4.06
Statistical significance	P value <0.05	Yes	Yes	Yes	Yes	Yes

**Figure 3:**
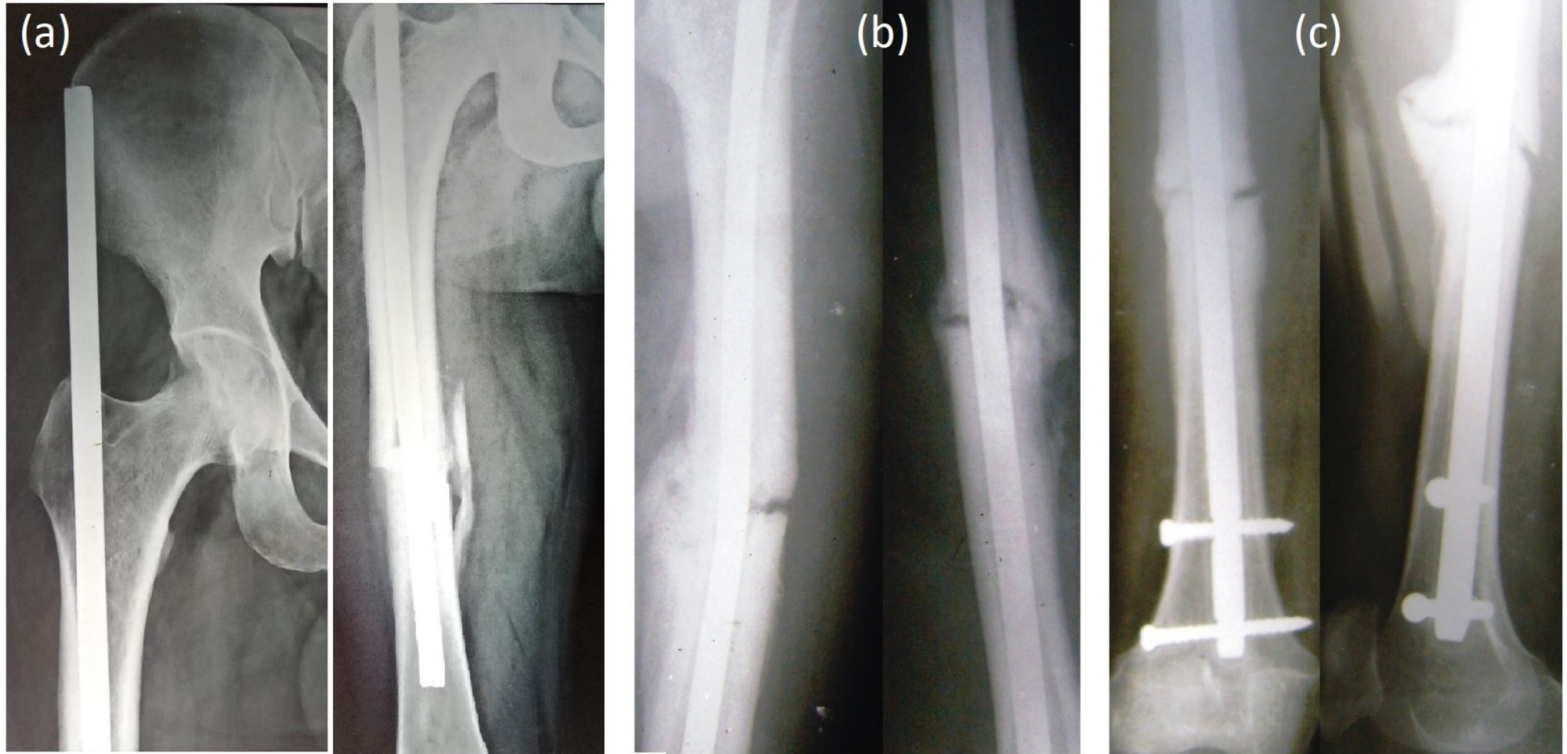
(a) Proximal migration of K-nail, (b) bent K-nail in group I. (c) Non-union in interlocking nailing in group II

Complications comprised of (Table 4, Figure 3) two cases of delayed union (4%) with implant failure (bent nail) and one case of implant migration in group I and one case in this group developed superficial postoperative wound infection which resolved after a course of antibiotics with no further evidence of deeper infection or osteomyelitis and no further intervention was required.

**Table 4:** Postoperative complications associated with K-nailing and intramedullary interlock nailing

Complications	Group I (K-nailing) (n = 25)	Group II (interlocking nailing) (n = 31)
Non-union	00	6.4% (2)
Delayed union	08% (2)	9.6% (3)
Implant failure (Including implant migration)	04% (1)	00
Infection	00	00
P value	0.192	

All three complicated cases in group I were treated by exchange nailing with thicker nail, and eventually all of them proceeded to union. There were two cases of non-union and three cases of delayed union in group II. The patients with non-union in this group were successfully treated with exchange nailing with thicker nail with cancellous bone graft, while the three patients with delayed union were treated by mere dynamization, which eventually got united. There was no implant failure in group II patients.

## Discussion

Interlocking intramedullary nailing has now become the implant of choice for the treatment of almost all types of fractures of the femoral shaft. Most of the centers routinely use ILNs to fix all types of femoral shaft fractures, including fractures of isthmic and paraisthmic zone. The use of unlocked intramedullary nail (K-nail) has virtually been abandoned.

Unlocked intramedullary nails were initially developed for fixation of transverse and short oblique fractures around the mid shaft region, i.e., the isthmic and paraisthmic areas of the shaft [11]. Nails with the diameter of the narrowest part of the medullary canal were usually used and they resisted rotational displacement by three-point fixation and friction. Interdigitation of the fracture ends provided further resistance against rotation. Later on, various studies proved the superiority of ILNs for shaft fractures except in the middle one-third shaft fractures. Therefore, K-nails were usually reserved for Winquist I and II femoral shaft fractures around the isthmus. The present study demonstrates that there is no significant difference in the outcomes between ILN and K-nail fixation of the patients when considering the union and the femoral alignment. The percentage of delayed union and non-union encountered in the group I and II was also not significant. Our major concern was the fact that group I patients had a higher rate of implant failure involving migration and bending of the nail. With our results, we concluded that the surgeons should probably reassess weight-bearing protocols when unlocked intramedullary nail fixation is employed and it seems that K-nail had an advantage over ILN when used for fracture around isthmus and paraisthmic area of diaphysis with the added advantage of less demanding surgery.

In our series, at sixth months we had union in 88% patients in group I and 84% patients in group II; patients in group I were walking without support by six months’ duration (with an average of 4.64 ± 0.54 months), whereas unsupported average walking time in cases of group II was 4.63 ± 0.60 months (p = 0.946) (Table 1).

We compared our results with studies of King et al. [12]. They reported 100% union at six months in 112 cases treated by K-nail. The infection rate was 1.7%. We had encountered two cases of non-union that were due to poor patient compliance. Similarly, Winquist et al. [13] reported union in 87% cases at three months amongst 520 cases and shortening of more than 2 cm in 20% cases managed by K-nail. Unsupported walking time was six weeks in 300 cases. Approximately 13% of cases landed in delayed union and non-union. Infection was reported in 9.6% of the cases. The results of their study are also comparable to the present series except for the infection which we did not encounter. The significant difference in unsupported walking time in our series was due to the reason that a large group of patients belonged to low socioeconomic status with less education. Therefore, it was judged that it is always better to wait for radiological fracture union before protected weight-bearing was allowed. Shortening was not a frequent complication encountered except in few cases (1-2.5 cm) of Winquist type II fractures: group I, three cases and group II, one case. We did not encounter any impacted nail, transient common peroneal nerve palsy, fat embolism, or any pulmonary complications in our cases.

Yu et al. [6] in their study of 93 cases of intramedullary nail fixation (43 cases using open K-nail and 50 with ILN) stated that there was no difference in the union rate between both groups (p = 0.3282). There were two cases of non-union and one case of delayed union in the K-nail group. There was one case of non-union and two cases of delayed union in the ILN group, which is comparable with the present study.

As a healthcare provider, our major concern is of exposure to ionizing radiation during surgery which is encountered as direct and scattered radiation from Image intensifier. Hak in his study stated that scattered radiation is the main source of radiation exposure for surgeons as well as for the patient. Out of around 1,000 photons emitted by intensifier, only 20 photons reach the detector, 100-200 photons, i.e. around 20% radiation, are scattered, and remaining photons are absorbed by the patient. Adverse effects of radiation on the body can be due to either somatic effects or stochastic effects. Somatic effects are directly related to the radiation dose. The dose of radiation required to produce radiation sickness is between 500 and 1,000 mSv. Early somatic effects include radiation sickness, whereas late somatic effects include leukemia, thyroid cancer, and radiation-induced cataracts so radiation exposure should be kept as low as reasonably achievable during nailing [14]. Fuchs et al. in their study found that during eight cases of closed ILN the average radiation dose received by the eye was 0.019 mSv, by the thyroid gland was 0.0354 mSv, and by the hands was 0.0417 mSv [15]. Our study clearly states that in comparison to K-nail, IMILN is associated with more radiation (Table 3) and maximum exposure to radiation occurs during distal locking [16]. With the advent of electromagnetic-assisted technique, radiation during distal locking has been effectively reduced in comparison to freehand fluoroscopy-assisted technique; moreover, electromagnetic-assisted technique is costly and also requires proper training in electromagnetic-assisted surgical techniques [17].

Thus, the complexity of implant and technique of IMILN increases the duration of surgery and radiation exposure. Therefore, they should also be given due importance and K-nail should be preferred over IMILN. As per the Indian scenario, we also found that K-nailing procedure is very cost-effective as we calculated the difference of financial burden on patients. The mean overall expenditure in K-nailing came out to be 1679 ± 0.80 Indian rupee, while it was 5032 ± 0.69 Indian rupee in ILN that was also statistically significant (p < 0.05).

Limitation of the study

The limitations in our study were small sample size and non-availability of radiation dosimeter to record scattered radiation from image intensifier.

## Conclusions

Kuntscher intramedullary nailing can provide comparable rates of union as is achieved with interlocking intramedullary nailing with an advantage of less radiation exposure and duration of surgery, provided the patient selection is proper (isthmic and paraisthmic zone). There is however a higher incidence of implant migration and bending and hypertrophic non-union associated with the use of K-nailing which occurred due to improper implant size selection. Considering the cost and surgical aspects and functional outcome, K-nail is still a viable option for a majority number of cases in many Indian hospitals, especially those with limited financial resources or less technical expertise.
